# Acute effects of beetroot juice and caffeine co-ingestion during a team-sport-specific intermittent exercise test in semi-professional soccer players: a randomized, double-blind, placebo-controlled study

**DOI:** 10.1186/s13102-022-00441-1

**Published:** 2022-03-29

**Authors:** Erfan Berjisian, Kerry McGawley, Bryan Saunders, Raúl Domínguez, Majid S. Koozehchian, Caio Victor Coutinho de Oliveira, Ramin Rafiei, Hossein Miraftabi, Amir Sarshin, Alireza Naderi

**Affiliations:** 1 Department of Exercise Physiology, Faculty of Physical Education and Sport Sciences, Tehran University, Tehran, Iran; 2 Swedish Winter Sports Research Centre, Department of Health Sciences, Mid Sweden University, Östersund, Sweden; 3 Applied Physiology and Nutrition Research Group, School of Physical Education and Sport, Rheumatology Division, Faculdade de Medicina FMUSP, Universidade de Sao Paulo, São Paulo, SP Brazil; 4 Institute of Orthopaedics and Traumatology, Faculty of Medicine FMUSP, University of São Paulo, São Paulo, Brazil; 5 Departamento de Motricidad Humana y Rendimiento Deportivo, Faculty of Education Sciences, Universidad de Sevilla, Sevilla, Spain; 6 Studies Research Group in Neuromuscular Responses, University of Lavras, Lavras, Brazil; 7 Department of Kinesiology, Jacksonville State University, Jacksonville, AL 36265 USA; 8 Medical Sciences Faculty, Facisa, Campina Grande, Paraíba Brazil; 9 Department of Exercise Physiology, Karaj Branch, Islamic Azad University, Karaj, Iran; 10 Department of Exercise Physiology, Borujerd Branch, Islamic Azad University, Borujerd, Iran

**Keywords:** Nitrate, Countermovement jump, Ergogenic aids, Team-sport performance, Yo–Yo test

## Abstract

**Background:**

Beetroot juice (BJ) and caffeine (CAF) are considered as ergogenic aids among athletes to enhance performance, however, the ergogenic effects of BJ and CAF co-ingestion are unclear during team-sport-specific performance. This study aimed to investigate the acute effects of BJ and CAF co-ingestion on team-sport-specific performance, compared with placebo (PL), BJ, and CAF alone.

**Method:**

Sixteen semi-professional male soccer players (age: 19.8 ± 2.2 years, body mass: 69.2 ± 6.1 kg, height: 177.3 ± 6.0 cm) completed four experimental trials using a randomized, double-blind study design: BJ + CAF, CAF + PL, BJ + PL, and PL + PL. Countermovement jump with arm swing (CMJAS) performance and cognitive function by Stroop Word-Color test were evaluated before and after the Yo–Yo Intermittent Recovery Test level 1 (YYIR1). Also, rate of perceived exertion (RPE), heart rate, and gastrointestinal (GI) discomfort were measured during each session.

**Results:**

No significant differences were shown between test conditions for total distance covered in YYIR1 (BJ + CAF: 1858 ± 455 m, CAF + PL: 1798 ± 422 m, BJ + PL: 1845 ± 408 m, PL + PL 1740 ± 362 m; *p* = 0.55). Moreover, CMJAS performance, cognitive function, and RPE during the YYIR1 were not significantly different among conditions (*p* > 0.05). However, the average heart rate during the YYIR1 was higher in CAF + PL compared to PL + PL (by 6 ± 9 beats/min; *p* < 0.05), and GI distress was greater in BJ + CAF compared to PL + PL (by 2.4 ± 3.6 a.u.; *p* < 0.05).

**Conclusion:**

These results suggest, neither acute co-ingestion of BJ + CAF nor BJ or CAF supplementation alone significantly affected team-sport-specific performance compared to the PL treatment.

## Background

Soccer is one of the most popular sports globally, and FIFA estimated a 34% increase in the number of participants between 2000 and 2011 [1]. Soccer players require high levels of aerobic fitness to cover large distances during match play (> 10 km) [2] while a cognitive component is also required due to decision-making during games [3]. Team-sport-specific tests such as Yo–Yo Intermittent Recovery Test 1 (YYIR1) and 2 (YYIR2) are used to simulate competition demands and evaluate intermittent running performance changes in team-sport athletes. Krustrup et al. [4] showed that performance in the YYIR1 test is significantly correlated (r = 0.81) with the number of high-intensity running actions performed at the end of each half of a game. The widespread use of nutritional supplements is commonplace within the sport to enhance athletic performance. While there is some evidence to support the use of selected performance supplements in specific soccer scenarios (e.g., creatine, beta-alanine, bicarbonate, caffeine, and nitrate/beetroot juice), there is a lack of information relating to the combination of these products and their combined ergogenic effects [5].

Caffeine (CAF) is a hugely popular supplement for soccer players aiming to improve performance [6]. Physiological responses following ingestion include increased catecholamine secretion, neuromuscular function, vigilance and alertness, and reduced perception of effort during exercise [7]. Some studies reported an improvement in total distance covered during soccer match-play [8], while others failed to show ergogenic effects of CAF on time to fatigue test in soccer players [9]. Nonetheless, CAF may be an effective supplement to improve soccer-specific exercise capacity.

Nitrate-rich beetroot juice (BJ) is another popular nutritional supplement which may be of interest for soccer. Specifically, improvements in team sport-specific tests like the YYIR1 have been shown with nitrate (NO3^−^) supplementation [10, 11], though some studies reported no improvement in team-sport high-intensity efforts during sport-specific actions following BJ ingestion [12, 13]. Mechanisms to explain these performance enhancements include increased blood flow, muscle contraction, calcium handling, and mechanical efficiency [14, 15]. Thus, BJ may also be an effective supplement to improve soccer-specific exercise capacity.

While the ergogenic effects of BJ and CAF may be of interest for team-sports athletes, information typically results from generic protocols that are isolated from other strategies that the athletes may also be implementing. In practice, athletes tend to consume several supplements simultaneously [5]. The combined use of supplements can occur either acutely, targeting a specific competition, or chronically throughout a training program. In this regard, previous studies have investigated the combination of BJ and CAF within a laboratory environment with contrasting results [16–19]. Cognitive function is essential for athletic performance with variables such as attention, memory, and executive functions involving working memory, decision-making, and multitasking shown to be improved by CAF and BJ ingestion separately [20, 21]. However, no studies have evaluated the combined effects of BJ and CAF on soccer-specific exercise capacity, lower limb muscular power measured by countermovement jump with arm swing (CMJAS), or cognitive performance. Given the independent beneficial effects that BJ and CAF may have on soccer-specific exercise capacity and their different physiological pathways, combining these two supplements may result in additive effects.

The current study aimed to investigate the isolated and combined effects of CAF and BJ supplementation on performance during the YYIR1, CMJAS, and cognitive function. We hypothesized that the increased CNS drive and reduced perceived exertion elicited by CAF supplementation, combined with improvements in metabolic efficiency resulting from BJ ingestion, would improve exercise capacity compared with taking each supplement in isolation or consuming a placebo (PL).

## Methods

### Participants

**Fig. 1:**
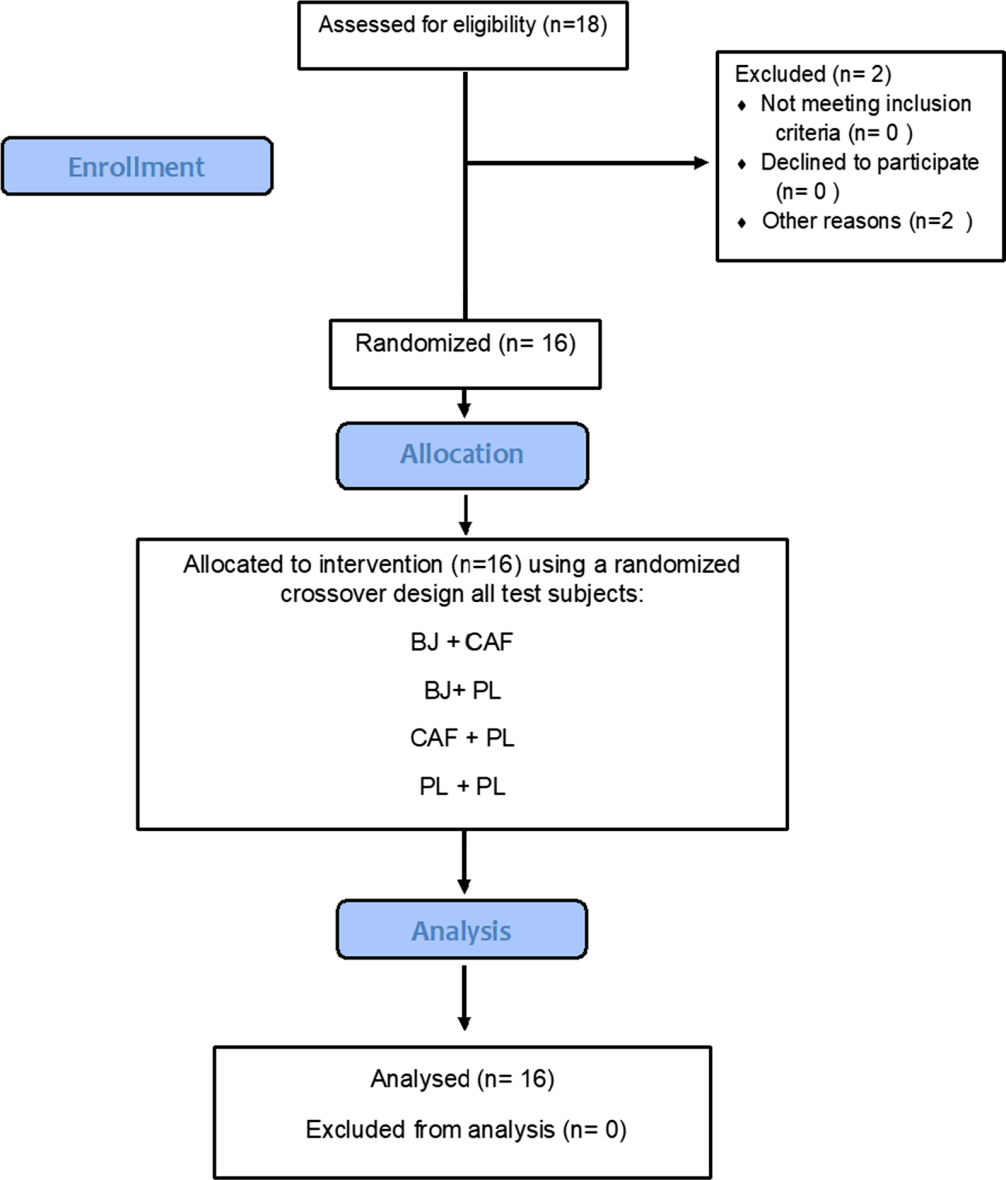
Participant allocation according to BJ + CAF, BJ + PL, CAF + PL, PL + PL. BJ, Beetroot juice; CAF, caffeine; PL, placebo

Eighteen semi-professional male soccer players from a competitive provincial soccer club volunteered to take part in the study. No participants reported any injury in the six months before the study or had used any ergogenic aids in the three months before the study. Participants completed a training history questionnaire, which revealed that they had been involved in soccer activity for ten years and trained five times each week (⁓ 9 h) during the investigation. Participants were informed of the potential risks, benefits, and dissemination of the research before providing written informed consent to participate. Tehran University Ethics Committee approved the study (IR.UT.SPORT.REC.1399.040) in accordance with the Declaration of Helsinki. Two participants failed to complete the study due to injuries unrelated to the protocol. Therefore, sixteen participants (age: 19.8 ± 2.2 y; body mass [BM]: 69.2 ± 6.1 kg; BMI: 22.1 kg/m^2^; height: 1.77 ± 0.06 m) completed the study (Fig. 1).

### Experimental design

**Fig. 2:**
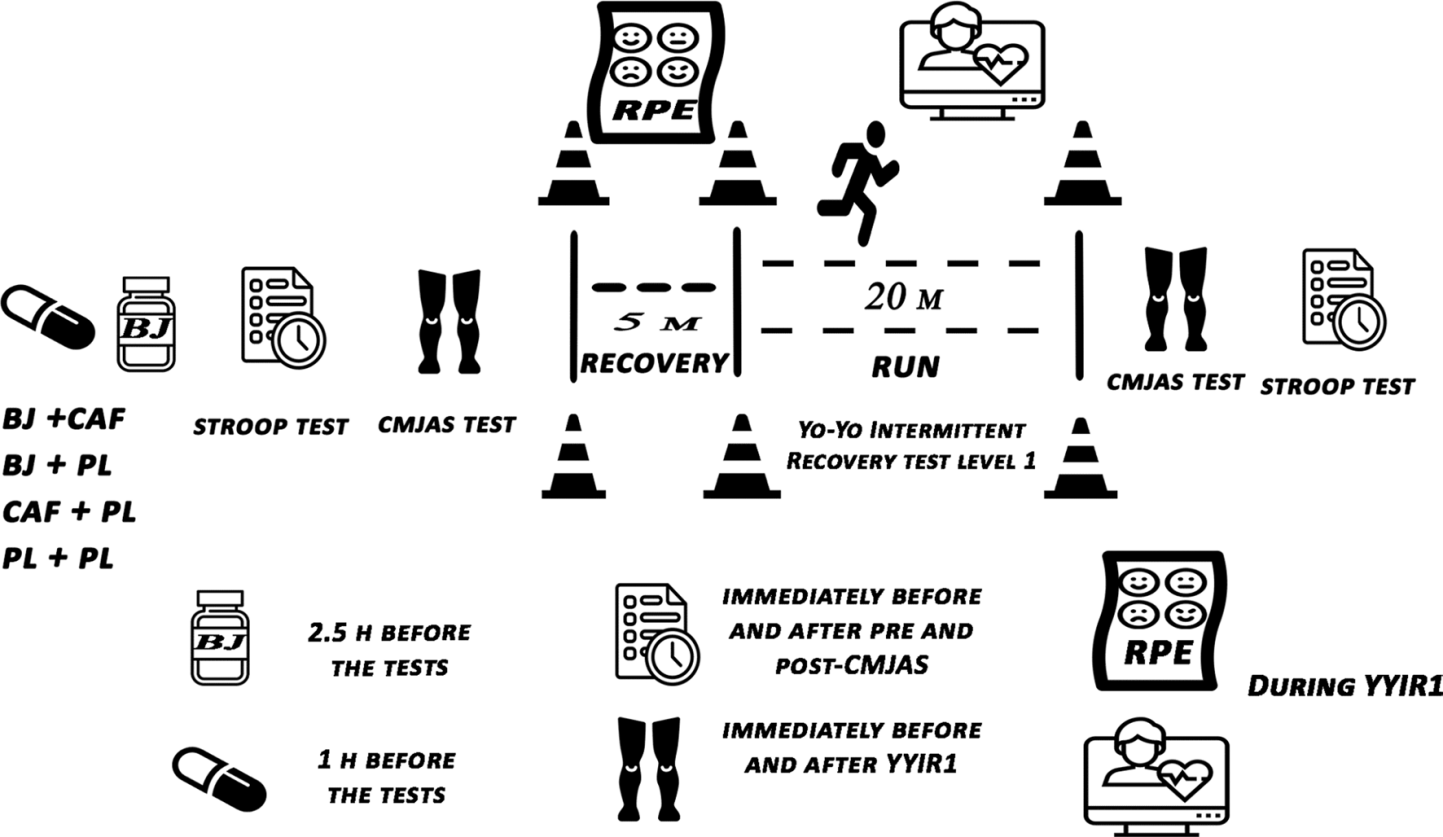
Schematic representation of the study design. BJ, Beetroot Juice; CAF, caffeine; PL, placebo; CMJAS, countermovement jump with arm swing, YYIR1, Yo–Yo intermittent recovery test level 1

The study followed a randomized, placebo-controlled, double-blind design (Fig. 1). Participants completed five test visits, including one familiarization session and four experimental trials with a one-week washout between each visit. During the familiarization, participants performed all aspects of the tests and procedures of the research. To avoid any influence from circadian variation as has been reported in previous studies, all trials were performed at the same time of day (11:00–13:00) for each participant [22, 23]. Our participants were not habituated to CAF (< 50 mg·day^−1^) as determined by a validated questionnaire [24]. Participants recorded 24-h food diaries before the familiarization session and were required to follow the same diet for 24 h before each experimental session and refrain from strenuous exercise for 48 h before each trial. This was checked by making a phone call before each session. Participants were given a list of NO3^−^-rich and caffeinated foods and drinks and were instructed to avoid consuming these items for 72 h before each trial. Furthermore, participants were asked to avoid using antibacterial mouthwash for 48 h before each trial to prevent the disruption of NO3^−^-reducing bacteria in the enterosalivary circulation [25] (Fig. 2).

### Supplementation protocols

Participants ingested one 60-mL bottle of fluid containing either 6.4 mmol (NO3^−^), 500 mg L-Arginine, and L-Ornithine (Red Beet Vinitrox Shot; Sponsor Ltd; Germany) or NO3^−^ depleted dried powder as a PL, from the same bottle 2.5 h before the start of the Stroop test. The PL was prepared by dissolving 1 g of dried powdered BJ in 1 L of mineral water and adding lemon juice to mimic the commercial supplement's taste. Even though the BJ present in the PL could have a minimum content of NO3^−^, the small proportion of desiccated BJ in the bottle of PL (0.015 g), along with the restricted intake of foods rich in NO3^−^ 72 h before the start of each session ensured that subjects working under the PL condition were depleted of NO3^−^ [26]. Participants also ingested a capsule containing 5 mg/kg BM of CAF (Cat. No. C0750; Sigma-Aldrich; Steinheim; Germany) or cellulose as PL 60 min before the start of the Stroop test. This resulted in four experimental trials consisting of BJ + CAF, CAF + PL, BJ + PL, and PL + PL. To ensure the double-blind study design, all supplements were randomized and blinded by an independent researcher not involved in the data collection. Participants were randomly assigned to each experimental condition using a Latin Square model [27] and Research Randomizer (www.randomizer.org). All participants were in a postprandial state before every test, having ingested a standardized breakfast of plain white bread and boiled eggs (1.5 g·kg^−1^ BM carbohydrate and 20 g protein) 3–4 h before the test.

### Exercise protocols

#### YYIR1

Following a 10-min standardized warm-up of stretching and jogging, participants started the YYIR1, which was performed indoors on a wooden surface within running lanes. Briefly, participants were required to perform 2 × 20-m shuttle runs repeatedly at a gradually progressive speed managed by bleeps provided by an audio system. Between every 2 × 20-m run, participants had 10 s of active recovery during which they walked around a cone placed 5 m behind the start/finish line [28]. A warning was given when a participant failed to cross the finish line before the bleep. When a participant was unable to cross the finish line before two consecutive bleeps, the last completed stage was registered, and the total distance covered was recorded as a measure of performance. Water was provided ad libitum throughout the rest period.

Heart rate (HR) was recorded continuously throughout the test using a standard monitor (Polar H10, Polar Team software, Kempele, Finland), with mean (HRav) and maximum (HRmax) values extracted as outcome variables, while the rating of perceived exertion (RPE; 6–20 scale) was recorded after every shuttle throughout the test. RPE data were subsequently averaged for every stage of the YYIR1 for statistical analysis.

#### CMJAS

Countermovement jump with arm swing (CMJAS) is suitable for soccer players and has been shown to generate ~ 15% greater jump height than CMJ in soccer players [29]. An experienced researcher informed participants of the proper CMJAS technique using video and live demonstrations during the familiarization session. Participants were instructed to stand with a straight torso and knees fully extended with the feet shoulder-width apart and then to perform a quick downward movement (to approximately 90° at the knees), followed by a fast-upward movement with the arms swinging back and forwards during the movement. Each participant practiced the CMJAS until the researcher was satisfied that the technique was correct. During the experimental trials, participants performed three CMJAS immediately before and after the YYIR1 using validated software (My jump2, https://www.carlos-balsalobre.com/), with approximately 30 s of rest between jumps. The highest jump was used for subsequent data analysis.

#### Cognitive function assessment

The Stroop Word-Color test was performed immediately before and after the pre-and-post-CMJAS, to determine any effects of supplementation and exercise on attention, interference, and cognition [30]. The test included a page composed of one hundred names of different color words printed in black ink (CW), a page containing one hundred Xs printed in different colors (C), and a final page with one hundred words from the first page printed in colors from the second page, where the ink color did not match the name of the color word (W). Participants read the words on the first page, the colors of the Xs on the second page, and the words on the third page (independent of ink color). Participants had to read each page aloud as quickly as possible within a fixed time of 45 s. The number of accurate responses attained on each page during the period was used to determine cognitive function. Each correct answer earned one point, and if the participant could repeat the pages within the period, the correct responses were included again. The first two pages evaluate congruence, while the third page evaluates incongruence or interference to determine the appropriate word, independent of the color [30].

#### Gastrointestinal discomfort

A questionnaire was used to evaluate any gastrointestinal (GI) discomfort symptoms 10 min before exercise [31]. This questionnaire included three sections, with each section containing four to seven questions, totaling 17 questions. Section one considered upper-GI issues, section two lower-GI issues, and section three any systemic problems like dizziness, headache, muscle cramp, and an urge to urinate. Each participant completed the 17 items on a 10-point scale ranging from 0 (no problems at all) to 9 (the worst it has ever been). Session data were grouped as the sum of scores for either upper-GI or lower-GI symptoms.

## Data analysis

Data were analyzed using the SAS statistical package (SAS® University Edition, SAS Institute Inc., USA) and are presented as mean ± SD. YYIR1 performance, HRav, HRmax, and GI discomfort were analyzed using a mixed model assuming supplementation (4 levels; BJ + CAF, CAF + PL, BJ + PL, and PL + PL) as fixed factors. The RPE was analyzed using a mixed model assuming supplementation (4 levels; BJ + CAF, CAF + PL, BJ + PL, and PL + PL) and time (12 levels [YYIR1 stages]) as fixed factors. CMJAS (maximum jump height and power output) and cognitive performance (CW, C, and W) were analyzed using a mixed model assuming supplementation (4 levels; as above) and time (2 levels; pre-and post- YYIR1) as fixed factors. Individuals were assumed as a random factor for all mixed models. Tukey–Kramer adjustments were performed when a significant *F* value was obtained. The data normality was determined using the Kolmogorov–Smirnov test and was confirmed for all variables except CW. These data were subsequently log-transformed and analyzed. Data for GI discomfort were also non-normally distributed and were analyzed using a non-parametric Kruskal–Wallis test. Hedge’s g effect sizes with a small sample size correction and 95% confidence intervals [CIs] were calculated for distance covered during the YYIR1, with minimum threshold values of 0.01, 0.2, 0.5, and 0.8 used to describe effect sizes as very small, small, moderate and large [32]. Results were interpreted according to the statistical probabilities of rejecting the null hypothesis (H0) in the following categories: *P* ≥ 0.1: no evidence against H0; 0.05 ≤ *P* < 0.1: weak evidence against H0; 0.01 ≤ *P* < 0.05: moderate evidence against H0; 0.001 ≤ *P* < 0.01: strong evidence against H0; *P* < 0.001: robust evidence against H0 [33].

## Results

### YYIR1

**Fig. 3:**
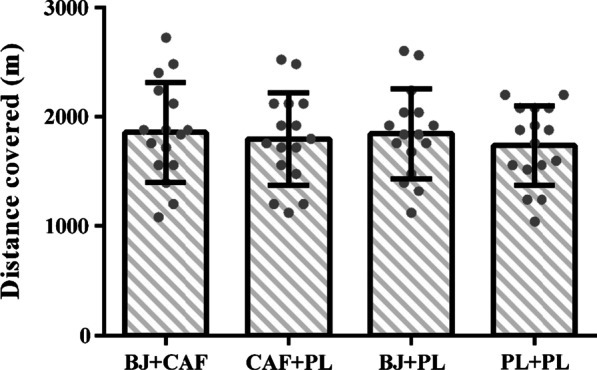
Mean ± SD for distance covered in the YYIR1 during the different supplementation sessions. Individual data are illustrated with grey dots. BJ, Beetroot juice; CAF, caffeine; PL, placebo

There was no evidence of a main effect of supplementation on total distance covered during the YYIR1 (F = 0.71, *p* = 0.55), which was 1858 ± 455 m in BJ + CAF, 1798 ± 422 m in CAF + PL, 1845 ± 408 m in BJ + PL and 1740 ± 362 m in PL + PL (Fig. 3). Group effect sizes ranged from very small to small (BJ + CAF vs. BJ + PL, g = 0.03 [95%CI: -0.66–0.72]; CAF + PL vs. BJ + PL, g = 0.11 [95%CI: − 0.58–0.80]; BJ + CAF vs. CAF + PL, g = 0.13 [95%CI: − 0.56–0.83]; CAF + PL vs. PL + PL, g = 0.14 [95%CI: − 0.55–0.84]; BJ + PL vs. PL + PL, g = 0.27 [95%CI: − 0.43–0.96]; BJ + CAF vs. PL + PL, g = 0.28 [95%CI: − 0.42–0.98]).

### Heart rate, RPE, and GI discomfort

There was moderate evidence of a main effect of supplementation on HRav (F = 3.59, *p* = 0.02), which was 175 ± 8 beats·min^−1^ in BJ + CAF, 177 ± 10 beats·min^−1^ in CAF + PL, 172 ± 10 beats·min^−1^ in BJ + PL and 171 ± 13 beats·min^−1^ in PL + PL. Post-hoc analyses showed moderate evidence of a difference between CAF + PL vs. PL + PL (*p* = 0.03). There was no evidence of a main effect of supplementation on HRmax (F = 0.43, *p* = 0.74), which was 198 ± 9 beats·min^−1^ in BJ + CAF, 200 ± 11 beats·min^−1^ in CAF + PL, 196 ± 10 beats·min^−1^ in BJ + PL and 196 ± 10 beats·min^−1^ in PL + PL.

There was no evidence of a main effect of supplementation on RPE (F = 0.45, *p* = 0.72) or of a supplement × time interaction effect (F = 0.47, *p* = 0.99). However, there was robust evidence of a time effect (F = 796.90, *p* < 0.001), with RPE increasing throughout the exercise (Table 1).

**Table 1:** Mean ± SD for the rating of perceived exertion (RPE) for every stage of the YYIR1

	BJ + CAF		CAF + PL		BJ + PL		PL + PL	
	RPE	N	RPE	N	RPE	N	RPE	N
Stage 9	6 ± 0	16	6 ± 1	16	6 ± 0	16	6 ± 1	16
Stage 11	7 ± 1	16	7 ± 1	16	7 ± 1	16	7 ± 1	16
Stage 12	7 ± 0	16	7 ± 1	16	7 ± 1	16	7 ± 1	16
Stage 13	8 ± 1	16	8 ± 1	16	8 ± 1	16	8 ± 1	16
Stage 14	10 ± 1	16	9 ± 1	16	10 ± 1	16	10 ± 1	16
Stage 15	12 ± 2	16	11 ± 1	16	12 ± 2	16	12 ± 1	16
Stage 16	15 ± 3	15	14 ± 2	16	15 ± 3	15	15 ± 2	15
Stage 17	17 ± 3	13	17 ± 2	13	17 ± 2	13	17 ± 2	12
Stage 18	19 ± 2	10	18 ± 2	9	19 ± 2	11	19 ± 1	9
Stage 19	19 ± 1	6	19 ± 1	5	18 ± 2	3	20 ± 0	5
Stage 20	20 ± 1	3	20 ± 1	2	20 ± 0	2	-	0
Stage 21	20	1	-	0	-	0	-	0

*BJ* Beetroot juice, *CAF* caffeine, *PL* placebo

There was no evidence of an effect of supplementation on upper-GI symptoms (*p* = 0.95), but there was moderate evidence of an effect on lower-GI symptoms (*p* = 0.02). The sum of scores showed moderate evidence for greater symptoms in BJ + CAF compared to PL + PL (2.4 ± 3.6 vs. 0.0 ± 0.0 a.u, respectively; *p* = 0.02), with no further evidence of differences. The sum of scores for CAF + PL was 0.9 ± 3.0 a.u. and for BJ + PL was 1.8 ± 3.8 a.u., and scores were generally low (range: 0–13).

### CMJAS

**Fig. 4:**
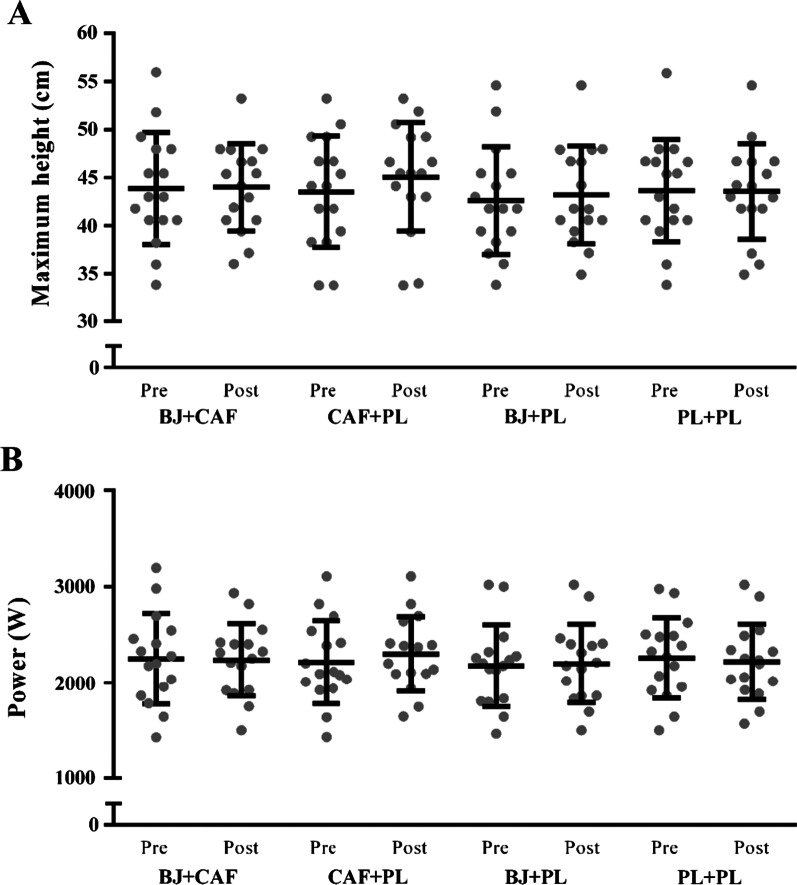
Mean ± SD for maximum countermovement jump with arm swing height (**A**) and power output (**B**) pre- and post-YYIR1 during each supplementation condition. BJ, Beetroot juice; CAF, caffeine; PL, placebo

There was no evidence of a supplementation effect (F = 0.21, *p* = 0.89) or supplementation × time interaction (F = 1.26, *p* = 0.30) on maximum CMJAS jump height (Fig. 4A). However, there was weak evidence for a main effect of time (F = 2.87, *p* = 0.09), with higher jumps recorded post-YYIR1. There was no evidence of an effect of supplementation (F = 0.09, *p* = 0.97), time (F = 0.61, *p* = 0.44), or supplementation × time interaction (F = 2.05, *p* = 0.12) on maximum CMJAS power output (Fig. 4B).

### Cognitive function

There was no evidence of a supplementation effect for Stroop test performance (CW: F = 1.10, *p* = 0.36; C: F = 0.31, *p* = 0.82; W: F = 0.38, *p* = 0.77), but there was strong evidence of a time effect (CW: F = 26.94, *p* < 0.001; W: F = 34.18, *p* < 0.001; C: F = 26.48, *p* < 0.001), with an increase in correct responses recorded post-YYIR1. There was weak evidence of an interaction for CW (F = 2.63, *p* = 0.06), with post-hoc analyses revealing very strong evidence of a difference from pre- to post-YYIR1 scores in CAF + PL (*p* = 0.0003; Table 2). By contrast, there was no evidence of a supplementation × time interaction for C (F = 0.44, *p* = 0.72) or W (F = 1.52, *p* = 0.22) (Table 2).

**Table 2:** Mean ± SD for number of correct responses during the Stroop test

	CW		C		W	
	Pre	Post*	Pre	Post*	Pre	Post*
BJ + CAF	120 ± 17	125 ± 14	97 ± 10	104 ± 14	127 ± 15	130 ± 15
CAF + PL	109 ± 15	123 ± 13*	94 ± 11	100 ± 14	121 ± 18	131 ± 14
BJ + PL	119 ± 18	127 ± 18	95 ± 16	104 ± 13	129 ± 18	135 ± 19
PL + PL	115 ± 15	118 ± 14	97 ± 14	102 ± 15	125 ± 15	135 ± 14

^*^Significantly different from Pre in the respective condition (*P* < 0.001)

*BJ* Beetroot juice, *CAF* caffeine, *PL* placebo

## Discussion

The current study investigated BJ and CAF supplementation effects on soccer-specific exercise capacity in isolation and combination. Contrary to the hypothesis, BJ + CAF co-ingestion did not lead to superior effects compared to the ingestion of either BJ or CAF alone or in comparison to a PL condition. Moreover, supplementation of both BJ and CAF in isolation did not lead to performance improvements compared to PL.

A growing body of scientific evidence supports the ergogenic benefits of BJ supplementation during high-intensity intermittent activities. Wylie et al. [10] showed a 4.2% improvement in YYIR1 in recreationally active soccer players after ingesting 29 mmol of NO3^−^ over a 30-h period compared to a PL (1704 ± 304 m vs. 1636 ± 288 m). Similarly, Thompson et al. [34] reported a 3.4% improvement in YYIR1 performance in male team-sport players after five days of ingesting 6.4 mmol of NO3^−^ (1422 ± 502 vs. 1369 ± 505 m). Nyakayiru et al. [11] also reported enhanced YYIR1 performance with BJ compared to a PL (+ 3.4%) in trained soccer players after six days of ingesting 12.9 mmol of NO3^−^. In contrast, our study showed that acute ingestion of 60 ml of NO3^−^-rich BJ containing 6.4 mmol of NO3^−^ 2.5 h before exercise did not improve YYIR1 performance in semi-professional soccer players. The training status of the participants could explain the difference between our results and others. Athletes here ran a higher distance in the test than those in previous studies [10, 11, 34], suggesting a superior training level. Based on the previous studies suggesting BJ is less effective in more trained individuals, it is possible that individuals with a higher aerobic fitness level need a greater amount of NO3^−^, perhaps via chronic supplementation, to sufficiently elevate intramuscular and plasma NO3^−^ to improve exercise performance [15, 35]. Thus, an insufficient dose could explain, at least in part, the lack of an effect shown here. Furthermore, we used an acute dose of BJ that may not have been sufficient to improve exercise capacity since previous studies showed improved YYIR1 performance following chronic supplementation, even though NO3^−^ doses > 5 mmol ingested approximately 2.5 h prior to exercise have previously been shown to exert ergogenic effects [15]. Furthermore, data suggest that the quantity of NO3^−^ found in some commercially available NO3^−^ products may not entirely represent the claimed values [36, 37]. Thus, the possibility that the NO3^−^ supplement used had a sub-optimal quantity of NO3^−^ cannot be entirely excluded. Our findings suggest that well-trained male team-sport athletes may require a chronic supplementation strategy and a higher total NO3^−^ dose to elicit ergogenic benefits, especially compared to that in recreational athletes [38], although this remains to be experimentally tested.

Caffeine is a popular ergogenic aid used extensively by team-sport athletes in doses ranging from 3 to 6 mg/kg BM [39]. However, equivocal findings are available concerning the efficacy of CAF administration on YYIR1 performance [40–42]. Ellis et al. [43] showed no improvement in YYIR1 performance in elite male soccer players following the ingestion of 1–3 mg/kg BM of CAF, and the authors suggested that a higher dose may be required to elicit performance gains. However, the present study does not support this suggestion since there was no significant improvement in YYIR1 performance following ingestion of 5 mg/kg BM of CAF in semi-professional soccer players. Therefore, the current findings add to the body of evidence indicating no clear benefit of CAF on YYIR1 performance. A recent meta-analysis by Grgic et al. [44] suggested that CAF improves YYIR2 performance, which may be due to the increased anaerobic system contribution. Inter-individual factors such as genotype variation, training status, and CAF tolerance have also been suggested to modify the erogenicity of CAF [45] and may have contributed to the results, although these factors were not explored in the current study.

This is the first study that investigated whether co-ingestion of BJ + CAF would be more effective than the ingestion of BJ or CAF alone during team-sport-specific exercise. In this regard, we hypothesized that the combination of these two supplements would be synergistic due to distinct, but potentially complementary, mechanisms. BJ was proposed to enhance the contractile function of type II muscle fibers and improve high-intensity intermittent performance [46]. Complementary to this, CAF could improve exercise capacity via its influence on the central nervous system which could delay fatigue [47]. However, the findings demonstrated no improvement during the YYIR1 test following combined- compared to single-supplement ingestion or PL. Effect sizes suggest that the co-ingestion of BJ and CAF led to a minimal increase in performance compared to CAF alone and compared to PL. Such an improvement may be worthwhile for soccer players, although confidence intervals were wide. Therefore, co-ingestion of these ergogenic aids before training and competition may be worth trialing on an individual basis. Finally, the lack of ergogenicity seen in these supplementation strategies may be related to external factors such as nutritional status (both acute and chronic), sleep pattern, physical activity level, training status, and small sample size [48]. Overall, this finding agrees with previous studies that reported no significant effects of combining BJ + CAF on maximal and submaximal running [19] or cycling [16–18].

There was moderate evidence for a higher HRav with CAF alone compared to PL, but not with the addition of BJ to CAF. This may be due to CAF ingestion's effects on the autonomic nervous system by increasing catecholamine secretion, which consequently increases HR [39], while the vasodilatory effects of BJ may eliminate this effect. RPE increased throughout the YYIR1 protocol but was not influenced by supplementation. This is in contrast to substantial data showing reduced RPE with CAF supplementation [49]. It is unclear why there was no effect in our study. In the current study, some participants reported low to moderate GI distress following BJ + CAF supplementation. The exact cause is unclear, and more studies are needed to confirm the safety and side-effects associated with BJ + CAF co-ingestion.

We showed no effect of supplementation on CMJAS jump height or power output. This result contrasts with a previous study that reported increased CMJ height after CAF supplementation [50, 51]. Previous studies reported no ergogenic effect of BJ on CMJ of professional tennis players or basketball players at the dose employed in this study (6.4 mmol of NO3^−^) [12, 13]. Although different studies have reported a reduced CMJ performance post-exercise that have proposed the diminution of height reached post-exercise as an indicator of neuromuscular fatigue [52, 53]; a trend to a higher height on CMJ has been found in this study. In this case, the higher jumps recorded post-YYIR1 could be related to post-activation performance enhancement during exercise running protocol that means the level of muscle potentiation is greater than neuromuscular fatigue associated with running exercise [54]. Furthermore, CMJ is considered a good indicator of lower limb muscular power, and additional transference of upper limb force can induce a greater jump height during the execution of a CMJAS, where the arms are involved [29]. This transference of force is dependent on good coordination to transfer angular moments between the different body segments. The effect of BJ supplementation may be less pronounced when the movement becomes more complex and involves more moving parts, although this hypothesis remains to be tested.

Cognitive ability, which plays an important role in team sports performance, includes the action of perception, learning, decision-making, and communication, and is sensitive to changes in physical demand, mood, and arousal [55]. At very high-intensity exercise (85%V̇O_2peak_) cognitive function deteriorates with a clear detrimental effect on reaction time [56]. Team sports players are generally required to make rapid and appropriate decisions whilst simultaneously exercising at variable intensities. There was an increase in correct responses during the Stroop test recorded post-YYIR1, consistent with previous studies suggesting that high-intensity intermittent exercise may benefit cognitive function [57]. While these benefits are well known, the mechanisms by which they occur are unclear [57]. However, supplementation with BJ and/or CAF showed no significant cognitive function effect pre-or post-exercise. These findings agree with previous studies that have investigated the isolated effects of BJ [58] or CAF [59].

This study has some limitations. It was not possible to measure blood NO3^−^, nitrite, or CAF concentrations, and the absence of these measurements might be considered the main limitation of the study. Similarly, we could not quantify the amount of NO3^−^ in the supplement provided, which may be more variable than claimed [37]. Furthermore, we did not perform a sample size calculation and chose to recruit a sample of convenience, which means our study might have been underpowered to detect small effects from supplementation protocols [60]. Also, blinding efficacy was not determined here; supplement identification could influence exercise outcomes and is a potential source of bias in sports nutrition [61]. The dosing strategy used here (an acute and low dose of BJ) is another possible limitation and it cannot be ruled out that greater or more chronic doses might lead to different results. Future research should consider these limitations to provide more insight into the co-ingestion of these ergogenic aids during team sport-specific performance.

## Conclusion

The results of this study showed that neither CAF, BJ, nor a combination of both elicits significant beneficial effects on the team-sport-specific intermittent running test. Soccer athletes and coaches should be aware that these supplements may not enhance soccer-specific exercise capacity or cognitive function. Future research may wish to determine whether a chronic BJ supplementation strategy is effective for soccer-specific exercise capacity, including soccer-specific tactical and technical tests, and whether these supplements are effective at improving YYIR2 performance.

## Data Availability

The current data in this study are available on request from the corresponding author.

## Abbreviations

BJ: Beetroot juice
CAF: Caffeine
PL: Placebo
YYIR1: Yo–Yo Intermittent Recovery Test level 1
YYIR2: Yo-Yo Intermittent Recovery Test level 2
CMJAS: Countermovement jump with arm swing
CMJ: Countermovement jump
RPE: Rate of perceived exertion
HR: Heart rate
HRav: Mean heart rate
HRmax: Maximum heart rate
GI: Gastrointestinal

## References

[1] Datson N, Hulton A, Andersson H, Lewis T, Weston M, Drust B (2014). Applied physiology of female soccer: an update. Sports Med.

[2] Barnes C, Archer D, Hogg B, Bush M, Bradley P (2014). The evolution of physical and technical performance parameters in the English premier league. Int J Sports Med.

[3] Rogan S, Schumacher N, Schmidt M, Wellmann K, Braumann K-M (2018). General perceptual-cognitive abilities: age and position in soccer. PLOS ONE..

[4] Krustrup P, Mohr M, Ellingsgaard H, Bangsbo J (2005). Physical demands during an elite female soccer game: importance of training status. Med Sci Sports Exerc.

[5] Naderi A, Earnest CP, Lowery RP, Wilson JM, Willems ME (2016). Co-ingestion of nutritional ergogenic aids and high-intensity exercise performance. Sports Med.

[6] Tallis J, Clarke N, Morris R, Richardson D, Ellis M, Eyre E (2021). The prevalence and practices of caffeine use as an ergogenic aid in English professional soccer. Biol Sport.

[7] Barreto G, Grecco B, Merola P, Reis CEG, Gualano B, Saunders B (2021). Novel insights on caffeine supplementation, CYP1A2 genotype, physiological responses and exercise performance. Eur J Appl Physiol.

[8] Del Coso J, Muñoz-Fernández VE, Muñoz G, Fernández-Elías VE, Ortega JF, Hamouti N (2012). Effects of a caffeine-containing energy drink on simulated soccer performance. PLOS ONE.

[9] Ferreira RES, Pacheco RL, de Oliveira-Cruz-Latorraca C, Riera R, Eid RG, Martimbianco ALC (2021). Effects of caffeine supplementation on physical performance of soccer players: systematic review and meta-analysis. Sports Health.

[10] Wylie LJ, Mohr M, Krustrup P, Jackman SR, Ermιdis G, Kelly J (2013). Dietary nitrate supplementation improves team sport-specific intense intermittent exercise performance. Eur J Appl Physiol.

[11] Nyakayiru J, Jonvik K, Trommelen J, Pinckaers P, Senden J, van Loon L (2017). Beetroot juice supplementation improves high-intensity intermittent type exercise performance in trained soccer players. Nutrients.

[12] López-Samanes Á, Pérez-López A, Moreno-Pérez V, Nakamura FY, Acebes-Sánchez J, Quintana-Milla I (2020). Effects of beetroot juice ingestion on physical performance in highly competitive tennis players. Nutrients.

[13] López-Samanes Á, Gómez Parra A, Moreno-Pérez V, Courel-Ibáñez J (2020). Does acute beetroot juice supplementation improve neuromuscular performance and match activity in young basketball players? A randomized, placebo-controlled study. Nutrients.

[14] Rojas-Valverde D, Montoya-Rodríguez J, Azofeifa-Mora C, Sanchez-Urena B. Effectiveness of beetroot juice derived nitrates supplementation on fatigue resistance during repeated-sprints: a systematic review. Crit Rev Food Sci Nutr. 2020;1–12. 10.1080/10408398.2020.1798351 32715742

[15] Senefeld JW, Wiggins CC, Regimbal RJ, Dominelli PB, Baker SE, Joyner MJ (2020). Ergogenic effect of nitrate supplementation: a systematic review and meta-analysis. Med Sci Sports Exerc.

[16] Lane SC, Hawley JA, Desbrow B, Jones AM, Blackwell JR, Ross ML (2014). Single and combined effects of beetroot juice and caffeine supplementation on cycling time trial performance. Appl Physiol Nutr Metab.

[17] Glaister M, Pattison JR, Muniz-Pumares D, Patterson SD, Foley P (2015). Effects of dietary nitrate, caffeine, and their combination on 20-km cycling time trial performance. J Strength Cond Res.

[18] Handzlik MK, Gleeson M (2013). Likely additive ergogenic effects of combined preexercise dietary nitrate and caffeine ingestion in trained cyclists. ISRN Nutr.

[19] Oskarsson J, McGawley K (2018). No individual or combined effects of caffeine and beetroot-juice supplementation during submaximal or maximal running. Appl Physiol Nutr Metab.

[20] Meeusen R, Decroix L (2018). Nutritional supplements and the brain. Int J Sport Nutr Exerc Metab.

[21] Lorenzo Calvo J, Fei X, Domínguez R, Pareja-Galeano H (2021). Caffeine and cognitive functions in sports: a systematic review and meta-analysis. Nutrients.

[22] Mora-Rodríguez R, Pallarés JG, López-Gullón JM, López-Samanes Á, Fernández-Elías VE, Ortega JF (2015). Improvements on neuromuscular performance with caffeine ingestion depend on the time-of-day. J Sci Med Sport.

[23] Dumar AM, Huntington AF, Rogers RR, Kopec TJ, Williams TD, Ballmann CG (2021). Acute beetroot juice supplementation attenuates morning-associated decrements in supramaximal exercise performance in trained sprinters. Int J Environ Res Public Health.

[24] Bühler E, Lachenmeier D, Schlegel K, Winkler G (2014). Development of a tool to assess the caffeine intake among teenagers and young adults. Ernahrungs Umschau.

[25] Easton C, Monaghan C, Liddle L, McIlvenna LC, Burleigh M, Muggeridge D, et al., editors. Anti-bacterial mouthwash reduces plasma nitrite following dietary nitrate supplementation but does not alter stress response. In: American college of sports medicine 64th annual meeting: 8th world congress on exercise is medicine® and world congress on the basic science of exercise and the brain; 2017.

[26] Domínguez R, Garnacho-Castaño MV, Cuenca E, García-Fernández P, Muñoz-González A, De Jesús F (2017). Effects of beetroot juice supplementation on a 30-s high-intensity inertial cycle ergometer test. Nutrients.

[27] Mason RL, Gunst RF, Hess JL (2003). Statistical design and analysis of experiments: with applications to engineering and science.

[28] Krustrup P, Mohr M, Amstrup T, Rysgaard T, Johansen J, Steensberg A (2003). The yo-yo intermittent recovery test: physiological response, reliability, and validity. Med Sci Sports Exerc.

[29] Mujika I, Santisteban J, Impellizzeri FM, Castagna C (2009). Fitness determinants of success in men's and women's football. J Sports Sci.

[30] Koozehchian MS, Earnest CP, Jung YP, Collins PB, O'Connor A, Dalton R (2017). Dose response to one week of supplementation of a multi-ingredient preworkout supplement containing caffeine before exercise. J Caffeine Res.

[31] Pfeiffer B, Cotterill A, Grathwohl D, Stellingwerff T, Jeukendrup AE (2009). The effect of carbohydrate gels on gastrointestinal tolerance during a 16-km run. Int J Sport Nutr Exerc Metab.

[32] Sawilowsky SS (2009). New effect size rules of thumb. J Mod Appl Stat Methods.

[33] Amrhein V, Korner-Nievergelt F, Roth T (2017). The earth is flat (*p* > 0.05): significance thresholds and the crisis of unreplicable research. PeerJ.

[34] Thompson C, Vanhatalo A, Jell H, Fulford J, Carter J, Nyman L (2016). Dietary nitrate supplementation improves sprint and high-intensity intermittent running performance. Nitric Oxide.

[35] Nyakayiru J, van Loon LJC, Verdijk LB (2020). Could intramuscular storage of dietary nitrate contribute to its ergogenic effect? A mini-review. Free Radic Biol Med.

[36] Shah I, Petroczi A, James RA, Naughton DP. Determination of nitrate and nitrite content of dietary supplements using ion chromatography. J Anal Bioanal Techn 2013;12(003).

[37] Gallardo EJ, Coggan AR (2019). What is in your beet juice? Nitrate and nitrite content of beet juice products marketed to athletes. Int J Sport Nutr Exerc Metab.

[38] Cermak NM, Gibala MJ, van Loon LJC (2012). Nitrate supplementation’s improvement of 10-km time-trial performance in trained cyclists. Int J Sport Nutr Exerc Metab.

[39] Guest NS, VanDusseldorp TA, Nelson MT, Grgic J, Schoenfeld BJ, Jenkins ND (2021). International society of sports nutrition position stand: caffeine and exercise performance. J Int Soc Sports Nutr.

[40] Abian-Vicen J, Puente C, Salinero JJ, González-Millán C, Areces F, Muñoz G (2014). A caffeinated energy drink improves jump performance in adolescent basketball players. Amino Acids.

[41] Ranchordas MK, King G, Russell M, Lynn A, Russell M (2018). Effects of caffeinated gum on a battery of soccer-specific tests in trained university-standard male soccer players. Int J Sport Nutr Exerc Metab.

[42] Muro N, Parada M (2016). Effect of caffeine on aerobic endurance performance. Mexican J Med Res.

[43] Ellis M, Noon M, Myers T, Clarke N (2019). Low doses of caffeine: enhancement of physical performance in elite adolescent male soccer players. Int J Sports Physiol Perform.

[44] Grgic J, Garofolini A, Pickering C, Duncan MJ, Tinsley GM, Del Coso J (2020). Isolated effects of caffeine and sodium bicarbonate ingestion on performance in the Yo–Yo test: a systematic review and meta-analysis. J Sci Med Sport.

[45] Pickering C, Kiely J (2018). Are the current guidelines on caffeine use in sport optimal for everyone? Inter-individual variation in caffeine ergogenicity, and a move towards personalised sports nutrition. Sports Med.

[46] Jones AM, Ferguson SK, Bailey SJ, Vanhatalo A, Poole DC (2016). Fiber type-specific effects of dietary nitrate. Exerc Sport Sci Rev.

[47] San Juan AF, López-Samanes Á, Jodra P, Valenzuela PL, Rueda J, Veiga-Herreros P (2019). Caffeine supplementation improves anaerobic performance and neuromuscular efficiency and fatigue in Olympic-level boxers. Nutrients.

[48] Esteves GP, Swinton P, Sale C, James RM, Artioli GG, Roschel H (2021). Individual participant data meta-analysis provides no evidence of intervention response variation in individuals supplementing with beta-alanine. Int J Sport Nutr Exerc Metab.

[49] Doherty M, Smith PM (2005). Effects of caffeine ingestion on rating of perceived exertion during and after exercise: a meta-analysis. Scand J Med Sci Sports.

[50] Lara B, Gonzalez-Millán C, Salinero JJ, Abian-Vicen J, Areces F, Barbero-Alvarez JC (2014). Caffeine-containing energy drink improves physical performance in female soccer players. Amino Acids.

[51] Lago-Rodríguez Á, Jodra P, Bailey S, Domínguez R. Caffeine improves performance but not duration of the countermovement jump phases. J Sports Med Phys Fitness; 2020. 10.23736/S0022-4707.20.11099-5 32720780

[52] Sanchez-Medina L, González-Badillo JJ (2011). Velocity loss as an indicator of neuromuscular fatigue during resistance training. Med Sci Sports Exerc.

[53] Maté-Muñoz JL, Lougedo JH, Barba M, García-Fernández P, Garnacho-Castaño MV, Domínguez R (2017). Muscular fatigue in response to different modalities of CrossFit sessions. PLOS ONE.

[54] García-Pinillos F, Ramírez-Campillo R, Boullosa D, Jiménez-Reyes P, Latorre-Román PÁ (2021). Vertical jumping as a monitoring tool in endurance runners: a brief review. J Hum Kinet.

[55] Hogervorst E, Riedel W, Jeukendrup A, Jolles J (1996). Cognitive performance after strenuous physical exercise. Percept Mot Skills.

[56] Fery Y, Ferry A, Hofe AV, Rieu M (1997). Effect of physical exhaustion on cognitive functioning. Percept Mot Skills.

[57] Calverley TA, Ogoh S, Marley CJ, Steggall M, Marchi N, Brassard P (2020). HIITing the brain with exercise: mechanisms, consequences and practical recommendations. J Physiol.

[58] Thompson C, Wylie LJ, Fulford J, Kelly J, Black MI, McDonagh ST (2015). Dietary nitrate improves sprint performance and cognitive function during prolonged intermittent exercise. Eur J Appl Physiol.

[59] Crowe MJ, Leicht AS, Spinks WL (2006). Physiological and cognitive responses to caffeine during repeated, high-intensity exercise. Int J Sport Nutr Exerc Metab.

[60] Lakens D. Sample size justification. 2021.

[61] Saunders B, de Oliveira LF, da Silva RP, de Salles PV, Gonçalves L, Yamaguchi G (2017). Placebo in sports nutrition: a proof-of-principle study involving caffeine supplementation. Scand J Med Sci Sports.

